# Barriers and facilitators to implementing staffing models in South African long-term care facilities: a qualitative study

**DOI:** 10.1186/s12912-025-03959-0

**Published:** 2025-10-24

**Authors:** Emerentia C. Nicholson, Mariana M. van der Heever, Cornelle Young, Anita S. van der Merwe

**Affiliations:** Department of Nursing and Midwifery, Stellenbosch University, Cape Town, South Africa

**Keywords:** Nurses, Staffing, Caregivers, Long-term care

## Abstract

**Background:**

South Africa has a staffing model with mandatory staffing standards for long-term care facilities for older persons. This article reports on the barriers and facilitators encountered during the implementation of the staffing model in long-term care facilities within resource-constrained contexts.

**Methods:**

A multiple-case study design was employed, comprising multi-methods for data collection, including interviews and a document review. This article concerns the findings from the interviews. Nineteen nurses and caregivers were purposefully sampled and interviewed using a semi-structured interview guide. Ethical and principles that were maintained included ethical board clearance, institutional permission, voluntary participation, beneficence, anonymity, and confidentiality. Trustworthiness was ensured through applying principles such as credibility, dependability, and confirmability. The data were analysed following a thematic analysis process and reported in narrative form. The findings are organised according to two themes: Human resource practices influence staff management, and A perceived indifference to and neglect of residents’ acuity.

**Findings:**

Facilitators in implementing the staffing model were high caregiver staffing levels and the availability of relief staff for caregivers. Barriers to implementing the staffing model included inadequate nurse staffing levels, the unavailability of relief staff for nurses, facilities’ neglect to provide in-service training, and an inadequate skill mix. The small number of nurses employed seemingly led to fewer nurses with higher educational levels and skills in the total staff mix and the overuse of caregivers.

**Conclusions:**

Policies should inform facilities’ staffing decisions to ensure adequate baseline nursing staffing and include support systems for nurses and caregivers to promote staff well-being.

**Supplementary Information:**

The online version contains supplementary material available at 10.1186/s12912-025-03959-0.

## Background

 Long-term care facilities (LTCFs) may provide services to older persons in resource-rich settings with sufficient access to human, economic, and social resources and infrastructure, or in resource-constrained settings with less access to these resources [1]. In South Africa, the income of private for-profit LTCFs tends to be higher than that of state-subsidised LTCFs. Private for-profit LTCFs receive monthly income from commercialising services to older persons for as much as R30,000 to R40,000 (approximately US$1,595 to US$2,126) [2]. State-subsidised LTCFs rely on monthly income from residents’ old age grants (state pensions) of R2,180 to R2,200 (approximately US$115 to US$116) at the time of writing [3].

Effective human resource management can help an organisation achieve its objectives. However, human resources comprise a considerable proportion of the healthcare budget [4]. Conversely, budget constraints and attempts to increase profits may result in LTCFs reducing nursing staff [5], thus favoring a lower-cost model of nurse categories. A shortage of RNs may also force LTCFs to employ lower categories of nurses [6]. Furthermore, the nurses’ scope of practice, which specifies the parameters within which each nurse category may provide services (Republic of South Africa, 2022), also influences the implementation of a staffing model in LTCFs. In South Africa, each of the nurse categories has its own scope of practice, which includes RNs (registered professional nurses providing comprehensive care and general registered nurses providing general care), enrolled nurses (ENs), with two years of training, and enrolled nurse assistants (ENAs), with one year of training. The ENs and ENAs have a limited scope of practice, which limits their utilisation in LTCFs, as they provide basic nursing care under direct or indirect RN supervision [7, 8]. However, the ENs as a nurse category is a legacy qualification being phased out, further increasing nurse shortages [9].

Employing less-skilled staff and fewer highly qualified nurses may lead to disparities in the skill mix, resulting in the overuse of less-skilled caregivers. South Africa has a prescribed staffing model that outlines the staffing standards LTCFs must apply according to residents’ acuity levels, including minimum staffing levels and skill mix [10]. As South African caregivers are not nurses but rather unlicensed staff who assist residents with daily activities [11], allocating most of the resident care to caregivers may result in the neglect of residents’ acuity. The extra workload of caregivers may overextend these health workers’ abilities when greater responsibilities, such as nursing responsibilities like medication administration, are assigned to them. Furthermore, South African LTCFs may also hesitate to use agency staff in emergencies, as the hourly tariff for agency RNs can be as high as R299,00 (approximately US$15,90). In comparison, caregivers receive about R28,79 (approximately US$1,53) per hour [12].

This study was part of a holistic multiple-case study within a larger PhD project conducted in one private for-profit and one state-subsidised LTCF in the Western Cape Province, South Africa. The holistic multiple-case study included a document review and interviews conducted simultaneously. The rationale for the study was that South African LTCFs experience insufficient staffing levels and incorrect skill mixes; however, there is limited evidence available on how LTCFs implement staffing models. This article reports on the findings of nineteen interviews to explore nurses’ and caregivers’ perspectives on the barriers and facilitators to implementing staffing models in the two selected LTCFs. The interviews were conducted from a critical realist perspective, allowing for a deeper exploration of the participants’ perspectives [13]. In addition to the meta-theory of critical realism, Mueller’s ‘Framework for Nursing Staffing in Long-Term Care Facilities’ was used to underpin the study due to its comprehensive perspective on critical concepts necessary for appropriate care in LTCFs. These critical concepts included staffing levels, skill mix, and staffing allocation in line with residents’ acuity levels, and the themes used in this study reflect these concepts [14].

## Methods

### Study design

The broader study design included a scoping review and a holistic multiple-case study. In the multiple-case study, we qualitatively explored nurses’ and caregivers’ views on perceived barriers and facilitators to implementing a nurse and caregiver staffing model in two LTCFs through interviews.

### Setting

The setting for all the interviews was one private for-profit (Code P1) and one state-subsidised LTCF (Code S2) in the Cape Metropole of the Western Cape province, South Africa. During data collection, the private for-profit LTCF provided accommodations for 35 residents, and the state-subsidised LTCF provided accommodations for 101 residents. P1 is located within a private retirement village, whereas S2 is a stand-alone facility.

### Population and sampling

The total study population in P1 included six RNs and 17 caregivers since this LTCF did not employ ENs and ENAs. The total study population in S2 included two RNs, seven ENs, three ENAs, and 25 caregivers. Through purposeful sampling and consideration of maximum variation, a diverse population was selected in terms of different nurse categories and caregivers (non-licensed workers), work experience, age, and gender (Grove & Gray, 2019). Appointments were scheduled with the LTCFs’ CEOs to meet and recruit potential participants. The inclusion criteria comprised all employed nurse managers, and RNs, ENs, ENAs, and caregivers who provided direct resident care. Potential participants were excluded from the study if they were on leave during the data collection period or were from personnel agencies due to their probable unfamiliarity with staffing issues in the LTCFs. All suitable participants received written invitations to voluntarily participate in the study. Table 1 provides a summary of the population and sample for the interviews.

**Table 1:** Population and sampling for interviews

Population	Private for-profit LTCF (P1) (30 to 40 beds)		State-subsidised LTCF (S2) (90 to 110 beds)	
	Target population	Sample	Target population	Sample
Nursing service manager/ facility manager	1	1	0	0
RNs	5	3	2	1
ENs	0	0	7	2
ENAs	0	0	3	3
Caregivers	17	5	25	4
**Totals**	**23**	**9**	**37**	**10**

### Data collection tool

A semi-structured interview guide was developed for the study (Supplementary File 1). It consisted of five open-ended questions related to the study’s critical concepts, i.e., staffing levels, skill mix, and staffing allocation aligned with residents’ acuity levels, ensuring that the researcher posed the same questions to all participants. A recording device was used to record audio during the interviews.

### Data collection

Appointments for interviews were scheduled with participants according to their preferred dates, times, and locations, while considering avoidance of service delivery interruptions. The primary researcher conducted all the interviews face-to-face in the two LTCFs between 9 March and 17 May 2023. Based on the participants’ preferences, fifteen interviews were conducted in English and four in Afrikaans. The interviews varied from 31 to 67 min, averaging 43 min. All interviews were recorded. The interviews continued until data saturation was reached, thus when new data contained redundant information [15].

### Trustworthiness

The ethical principle of practicing beneficence was maintained, and participants were assured of confidentiality. Code names were assigned to interview transcripts, recordings, and the LTCFs to ensure their anonymity. Two of the co-authors physically observed three random interviews and co-checked three to six transcriptions to facilitate credibility. The verbatim transcriptions were provided to participants for verification and to allow for corrections.

### Data analysis

After the interviews, the audio recordings were downloaded onto a computer and stored on a USB device for archive purposes. The primary researcher transcribed the interviews verbatim onto Microsoft Word documents in English or Afrikaans, depending on the language used during the interviews. Responses in Afrikaans were translated into English. All data were uploaded to ATLAS.ti for data management and qualitative analysis. The inductive thematic analysis approach, as described by Braun and Clarke (2006), was used to analyse the data as it aligned with the meta-theory of critical realism. Accordingly, to become familiar with the data, the audio recordings were re-listened to, and the transcripts read and re-read. Initial codes were then created for portions of the data as part of the process of searching for themes by grouping data with similar meanings. Redundant codes were set aside, reviewed, and the final themes appropriately labelled [16]. Theme saturation was reached, as the themes covered the range of data, participants’ perspectives, and the study’s central concepts [17].

## Findings

The final sample included 19 interviews. The nine interviews conducted in P1 included one facility manager (who was also the RN in charge), an additional three RNs, and five caregivers. No ENs or ENAs could be interviewed since P1 did not employ these nursing categories. All the participants were female since they employed no males. The ten interviews conducted in S2 included one RN, two ENs, three ENAs, and four caregivers, of whom one was male and nine were female. The findings include verbatim quotes that reflect participants’ viewpoints and are organised according to two themes: Human resource practices influence staff management, and A perceived indifference to and neglect of residents’ acuity. Table 2 displays the themes and subthemes derived from the interviews.

**Table 2:** Thematic presentation of themes derived from the interviews

Themes	Subthemes	Examples of quotes
Human resource practices influence staff management	Contextual factors influence nurse and caregiver staffing in the LTCF	There were disciplinary on top of disciplinary… it makes you unhappy. You come to work, then you ask yourself what will happen again today. You work under such stress. (Caregiver 2, S2).
	Managing nurse and caregiver fluctuations	I didn’t ask [for a replacement]. Because I know the question will be money. The answer is always money. (RN 9, S2).
	Low nurse and higher caregiver staffing	If I’m really busy or unable [to administer medication], she (a caregiver) has been here for 32 years, and I can trust her [handing the medication trolley’s keys to the caregiver during the interview]. I know she can read and see and think. (RN 17, P1).
	The available knowledge and skills of the nurses and caregivers	She [referring to a resident’s daughter] took her mother out here at [the name of S2] and put her mother in the [name of another suburb], somewhere that specialised in Alzheimer’s. Because she says, she doesn’t feel we have the knowledge or anything like that… We are not sent for training on how to handle a Dementia person or how to handle an Alzheimer’s person… (ENA 6, S2).
A perceived indifference to and neglect of residents’ acuity	Aligning nurses’ and caregivers’ skills with residents’ needs	When I come in, the first thing I do is prepare tea. Because there’s nobody making tea at night. In fact, it’s not as if I invented that because there’s no point in making tea [as a trained registered nurse]. (RN 18, P1).
	Overburdening the caregivers and the influence on residents	But colostomy bags are not the carer’s [caregiver’s] job to… It is not our scope of practice… we only learn it. But then there are some of the staff nurses [ENs] who say you MUST do it. It is expected of you. (Caregiver 2, S2).

### Human resource practices influence staff management

Although a mandatory requirement in the South African Regulations Regarding Older Persons, 2010, formal staff meetings were not held in these two LTCFs. Participants in S2 perceived the informal communication as filled with threats towards the staff:

*That’s what [facility manager’s name] told us again*,* I think it was last month*,* [the manager’s name] [says] I am not gonna get a replacement for you; otherwise*,* you’re not gonna get bonus at the end of the year …. We have to work like that. We have to accept it. If we don’t accept it*,* take your things. You can go because you’re not chain-bound….* (ENA 6, S2).

Disciplinary measures against staff were seldom required in P1 and were managed informally. In contrast to P1, disciplinary measures were frequently initiated in S2, causing discomfort among the nurses and caregivers:

*There were disciplinary on top of disciplinary: little things*,* unnecessary things. And I mean*,* it makes you unhappy. You come to work*,* then you ask yourself what will happen again today. You come in with that attitude: Oh*,* my word*,* what’s waiting for me again? You work under such stress. Because if you just hear that one’s are disciplined*,* those ones are disciplined… and that’s not nice.* (Caregiver 2, S2).

Relief staff were supplied with instances of caregiver absences in both LTCFs. However, S2 struggled to provide relief staff for nurses, ostensibly due to financial constraints, resulting in a reluctance to request relief staff:

*I didn’t ask [for a replacement]. Because I know the question will be money. The answer is always money.* (RN 9, S2).

According to the South African standards for the number of frail residents, P1 was required to provide a minimum of 11 nurses and five caregivers, and S2 had to provide a minimum of 15 nurses and nine caregivers [10]. Both LTCFs appeared to augment the minimum staffing levels by increasing the number of caregivers (lower cost) rather than nurses (higher cost). P1 provided four nurses and 18 caregivers, and S2 provided 12 nurses and 25 caregivers. The nurse-to-resident ratio was 1:35 in P1 and between 1:33 and 1:101 in S2, depending on how many nurses were on duty. P1’s RNs’ day duty work hours were reduced from 12 to 9 h due to budget constraints; therefore, there were three hours daily when no nurses were on duty. RNs managed the nurse shortfall of these three hours through staying on duty when the need arose:

*But if there is anything*,* like sometimes you see that a person is on oxygen or one of that*,* you wait*,* maybe there’s a change of condition*,* then I won’t leave till the sister [RN] is here.* (RN 18, P1).

The caregiver levels were considerably higher than the nurse levels in both LTCFs. With 18 caregivers in P1, the caregiver-to-resident ratio was 1:7 during the day and 1:11–12 during the night. With 25 caregivers in S2, the caregiver-to-resident ratio was between 1:11 and 1:12 during the day and 1:25 at night. With fewer nurses and more caregivers, participants still considered the caregiver staffing levels inadequate but appreciated the caregivers’ assistance since it alleviated the nurses’ workloads:

*You can rely on them … they know how to do their washes…we need more carers [caregivers]. I don’t have to go back and check on those small little things that must be done….* (ENA 4, S2).

According to the South African standards for the number of frail residents, P1 had to provide a minimum skill mix consisting of 16.75% RNs, 16.75% ENs, 33.25% ENAs, and 33.25% caregivers [10]. However, P1 provided 18.1% RNs and excluded ENs and ENAs, and 81.8% caregivers. S2 had to provide a minimum skill mix consisting of 12.5% RNs, 12.5% ENs, 37.5% ENAs, and 37.5% caregivers [10]. Yet, S2 provided 5.4% RNs, 18.9% ENs, 8.1% ENAs, and 67% caregivers. Higher responsibilities were seemingly shifted to less-trained nurses, such as using RNs and ENs interchangeably despite differences in these nurse categories’ scope of practice:

*When the sister [RN] is there*,* the sister will see to that all the areas is covered with staff and*,* uhm*,* yeah*,* the basic managing of the staff. The staff nurse [EN] would do medication and sometimes must even take charge. And yeah*,* help the sister*,* or if the sister is not in*,* they will be taking on the role of the sister [RN].* (Caregiver 8, S2).

With low proportions of nurses versus higher proportions of caregivers, higher responsibilities were also assigned to caregivers:

*If I’m really busy or unable [to administer medication]*,* she has been here for 32 years*,* and I can trust her [handing the medication trolley’s keys to the caregiver during the interview]. I know she can read and see and think.* (RN 17, P1).

Formal in-service training was not provided at the LTCFs. Participants in S2 shared that they needed training in managing residents with Alzheimer’s and Dementia:

*She [referring to a resident’s daughter] took her mother out here at [the name of S2] and put her mother in the [name of another suburb]*,* somewhere that specialised in Alzheimer’s. Because she says*,* she doesn’t feel we have the knowledge or anything like that… We are not sent for training on how to handle a Dementia person or how to handle an Alzheimer’s person…* (ENA 6, S2).

### A perceived indifference to and neglect of residents’ acuity

No staff-to-resident allocation was documented in P1. Caregivers could independently decide which residents to attend to per shift, adding a burden to the caregivers, possibly beyond their capabilities, and without the caregivers fully understanding the accompanying clinical and legal implications:

*They [the caregivers] arrange among themselves. … we [the caregivers] are in room five today*,* and room four… The other two [caregivers] do the other rooms. Tomorrow*,* they [the caregivers] exchange again. So*,* I don’t know how they do it*,* but that’s how they do it… to write a book: you do this and that*,* is time-consuming*,* they [the caregivers] don’t read it.* (RN 17, P1).

The allocation strategy used in S2 was to indicate in the duty rosters the area in the building where the staff had to work for approximately three months:

*On the duty roster in the beginning of the month*,* we allocate the staff like in the A passage*,* the D and E ward and the B and C ward. They stay like three months in an area so that they don’t get over tired of the area.* (RN 9, S2).

Although the RNs in both LTCFs seemingly worked within their scope of practice, the RNs on night duty in P1 were required to perform non-nursing tasks in the absence of domestic staff:

*When I come in*,* the first thing I do is prepare tea. Because there’s nobody making tea at night. In fact*,* it’s not as if I invented that because there’s no point in making tea [as a trained registered nurse].* (RN 18, P1).

Most direct resident care was assigned to caregivers, who were seemingly frustrated and overburdened due to high workloads:

*For instance*,* [a resident’s name]: she asked me for the pan*,* I give the pan to her. Then [another resident’s name] calls; she wants to go to the toilet. Then I put (the second resident’s name) on the toilet. I said mam*,* I’m coming back. I just going to take a pan there. So*,* then (a third resident’s name) also calls*,* and then I must run. I must take her also to the toilet. And then*,* eventually*,* I must go to [the first resident’s name] because I put her on the pan… but I cannot divide myself into three parts!* (Caregiver 5, S2).

Moreover, nursing tasks were shifted to caregivers beyond their job descriptions. They were seemingly also deprived of the opportunity to decline or question the validity of an assignment, further increasing the burden on the caregivers:

*But colostomy bags are not the carer’s [caregiver’s] job to… It is not our scope of practice… we only learn it. But then there are some of the staff nurses [ENs] who say you MUST do it. It is expected of you.* (Caregiver 2, S2).

The participants in both LTCFs alluded to delaying care, such as deferring communication with residents and omitting activities like nail care. No adverse events were documented in P1, but the document review findings revealed that S2 recorded various adverse events.

## Discussion

The implementation of staffing models was viewed through the lens of critical realism. It allowed for a deeper exploration of the complex underlying causal mechanisms and unobservable social realities influencing staffing [13] and provided a better understanding of the practices embedded in implementing a staffing model. The findings are discussed according to the themes in Table 2.

Various human resource practices influenced nurse and caregiver staffing in the two LTCFs, such as poor communication, disciplinary practices, and the provision of relief staff in instances where staff were absent. Complex social realities, such as social relationships and the use of power, could also shape reality [13]. For instance, neglecting to hold staff meetings and threatening staff when communicating informally may create a hostile work environment. Workplace hostility may demotivate staff, leading to job dissatisfaction and increased intentions to leave the workplace [18]. In contrast, managers who value inclusion and embrace open communication create work environments where staff are allowed to flourish [19]. The participants’ viewpoints were that disciplinary measures in the state-subsidised LTCF were excessive and stressful, which influenced their well-being. These perspectives aligned with literature, which indicated that healthcare professionals who were exposed to disciplinary measures experienced stress and depression [20]. Although disciplinary measures are intended to improve staff performance, job satisfaction can decrease when management fails to provide feedback on employee performance, leading to further poor performance [21].

The LTCFs managed staff absenteeism by providing relief staff for caregivers, but seldom for nurses. In the light of financial constraints, LTCFs often fail to budget for sufficient and professional staff [22]. The absence of relief staff contributes to higher workloads and increased responsibilities for the remaining nurses and caregivers, impeding their health, even more absenteeism [23], and impaired judgment leading to potential errors [24].

Higher total staffing levels may result in fewer falls among residents [25], fewer use of restraints, and reduced hospitalisations [4], and in fewer resident deaths [26]. In addition, residents’ activities of daily living may improve [27]. Compared to higher staffing levels, low staffing levels may increase workloads and lead to lower job satisfaction among the staff, resulting in burnout [22]. Various other studies found similarly low nurse staffing levels in the overall staffing, especially the RN staffing levels [27, 28]. In contrast to the low nurse staffing levels, the caregiver staffing levels were higher, with lower caregiver-to-resident ratios, apparently compensating for the low nurse staffing levels. Other studies confirmed that caregivers provided more hours of care per resident day than nurses [22, 27, 29].

The low proportion of nurses versus caregivers in this study led to an overuse of caregivers and disparities in the skill mix, with less skilled nurses available to care for residents with higher acuity levels. Moreover, the failure to provide in-service training in this study seemingly led to a perceived lack of knowledge regarding care provision to residents with Alzheimer’s or Dementia. An experimental study conducted in two Chinese nursing homes found that the experimental group, which received intensive training in legislation, ethics, and clinical competence, had a significantly higher competency score compared to the control group, which received standard training. Residents were also considerably more satisfied with the services provided by the experimental group [30]. Various studies supported the notion of a higher skill mix; thus, higher-skilled nurses in the total staffing, leading to an improvement in the quality of care [4, 27, 31]. When the skill mix is diluted by increasing the number of less-qualified staff, such as licensed practice nurses, nursing assistants, or unlicensed staff, it may lead to a decrease in the quality of care [4]. Moreover, the impact of low staffing levels and an insufficient skill mix was shown in a litigation case study using data from a court case against a private for-profit LTCF chain from the end of 2006 to the middle of 2009 in the United States. During the above-mentioned timeframe, residents and families lodged more than 3,000 grievances against 12 LTCFs in this private for-profit LTCF chain, of whom approximately 700 grievances concerned low staffing levels and an insufficient skill mix, resulting in high financial penalties for the private for-profit LTCF chain [32].

South African legislation requires LTCF staff to identify residents’ needs for professional and skilled care, such as pressure care and specialised wound care, assistance with incontinence, and support with mental functioning. Residents’ dependency needs must also be assessed to determine the staff assistance required during daily living activities [10, 33]. Similarly, countries like Germany and Australia conduct comprehensive individual assessments to determine residents’ acuity levels and the demand for care [34]. Based on residents’ acuity levels, the intensity of nursing care or the demand for care is determined [35]. However, staff allocation was not documented, staff were allocated according to the building layout regardless of the residents’ needs, or caregivers could decide independently which residents to attend to per shift. These findings align with those of other studies, indicating that LTCFs did not employ acuity-based staffing methods [27, 36].

Furthermore, there seemed to be an indifference to residents’ acuity with staff allocations. Mueller (2000) suggested that nurse managers consider legislative directives, the nurses’ scope of practice, policies, and job descriptions before assigning staff to ensure the staff are competent to complete the assigned tasks. Regarding the nurses’ scope of practice, RNs and certified nurse assistants may be used interchangeably in South Korea [37, 38] despite RNs typically having higher clinical decision-making skills and being equipped to provide higher-level resident care [39]. However, the interchangeable use of RNs and ENs is not allowed in South Africa due to their varying scope of practice. For example, ENs may only administer medication under RN supervision [7, 8]. Thus, one can assume that there may be legal implications for the EN participants and the state-subsidised LTCF where ENs performed tasks beyond their scope of practice.

Despite varying scopes of practice, task shifting occurred from higher-trained to less-trained nurses, and from nurses to caregivers. A deeper exploration of the influence of this task shifting showed that the caregivers had to assume nursing responsibilities beyond their competency levels and which increased their workloads. A study conducted in New Zealand on caregivers’ work stress in LTCFs found that caregivers had physically demanding work, and their stress levels were increased by a lack of recognition and feelings of not being heard [40]. Despite low nurse staffing levels in this study, nurses were required to perform non-nursing tasks. A cross-sectional study in Italy, including 743 nurses in 54 nursing homes, found that their participants spend 37% of their time on administrative tasks such as answering phone calls and scheduling appointments [41]. A study in Switzerland found that RNs spend only about 32% of their time on patients and do not work to their full scope of practice [42].

When allocating staff, indifference to residents’ acuity may lead to delayed or omitted care and adverse events. A previous study across 13,533 LTCFs in the United States reported that 22% of care was omitted in residents with higher acuity levels [43]. In another study, researchers analysed 1560 adverse event reports in ten Australian nursing homes. The findings showed that 18.5 adverse events occurred per nursing home per month during the day shifts and increased to 33.5 per nursing home per month during the night shifts [44]. Authors suggested that implementing acuity-based standards in LTCFs, thus determining staffing in LTCFs based on residents’ acuity levels, may improve residents’ outcomes [27, 36]. Although the South African minimum mandatory staffing standards for LTCFs are based on residents’ acuity levels, these standards appear insufficient to provide adequate resident care and need revision. The mandated amount of care a frail South African resident receives is 2.57 h per resident day (HPRD) [10], which falls short of the 4.1 HPRD recommended by researchers [5]. In fact, different provinces and states within countries often set higher staffing standards than those mandated by federal or provincial governments [36, 45]. It is also advised that LTCFs proportionally increase the skill mix as staffing levels rise.

### Strengths and limitations

The participants’ perspectives provided valuable insight into the perceived barriers and facilitators to implementing a staffing model in the LTCFs. However, only 19 interviews were conducted due to the low nurse staffing levels at the LTCFs and the exclusion of nurse categories (ENs and ENAs) in the private for-profit LTCF. Additionally, interviews were not conducted with the CEOs of the LTCFs, who could have provided valuable insights into budget decisions. Transferability to other contexts might be limited because it included only two LTCFs in the Western Cape province.

## Conclusion

This study was among the first to report nurses’ and caregivers’ perspectives on barriers and facilitators to implementing a staffing model in South African LTCFs. The findings suggested an underemployment of all categories of nurses and an overemployment of caregivers, leading to disparities in skill mix and residents receiving the same basic care regardless of their individual needs. More research should be conducted in Africa, specifically Sub-Saharan African LTCFs, focusing on whether nurse deficiencies are linked to adverse events. Policies should inform staffing decisions to ensure adequate baseline nursing staffing and include support systems for nurses and caregivers to promote staff well-being. Additionally, policy initiatives should consider a balance between cost-effective staffing and adverse resident outcomes.

## Supplementary Information

Below is the link to the electronic supplementary material.

Supplementary Material 1

## Data Availability

The datasets supporting the conclusions of this article are included within the article.
