# Supplementary material for: Barriers and facilitators to implementing staffing models in South African long-term care facilities: a qualitative study
Source: BMC Nurs. 2025 Oct 24;24:1326. doi: 10.1186/s12912-025-03959-0 (PMC12553242; doi:10.1186/s12912-025-03959-0)
Supplement: Supplementary file 1 — Supplementary Material 1 [file 12912_2025_3959_MOESM1_ESM.docx]

supplementary file 1

Interviews - Semi-structured interview guides

**Nurses (nursing service manager, registered, enrolled, and assistant nurses)**

**Title:** Developing a framework to inform staffing models for long-term care facilities in resource-constrained contexts.

**Introduction:** Through the years of coming in close contact with long-term care facilities, I became interested in how the legally prescribed staffing model is implemented in the facilities. Literature showed that staffing shortages in LTCFs occur worldwide and may be influenced by the higher demand for health care due to the growing number of older people. Therefore, the interview guide questions will explore the barriers and facilitators to implementing the staffing model in the LTCFs regarding staffing levels, skill mix, and staff allocation aligned with residents’ acuity. Your input will be valuable in developing a framework to inform staffing models for long-term care facilities in resource-constrained contexts.

**Questions**

1. Please tell me how you experienced implementing the staffing model in your LTCF regarding the number of nurses and caregivers employed?

**Probing words:** balance between nurses and caregivers, decision making, resident outcomes

1. In your experience, what aspects increase the ability of your LTCF to provide/ensure enough nurses and caregivers?
2. In your experience, what aspects decrease the ability of your LTCF to provide/ensure enough nurses and caregivers?

**Probing words (questions 2 and 3):** recruitment, appointment practices, absenteeism, staff turnover, availability of temporary staff, efforts to adjust staff totals during sick and vacation leave periods

1. Tell me your experience/view on how the nurses and caregivers are allocated to residents or tasks and how the skill mix influences the provision of care?

**Probing words**: who is responsible for the allocation, type of allocation, reasons/bases for distribution, rotating between residents, residents’ acuity, efforts to adjust per residents’ acuity, task allocation, consideration of the scope of practice and skill mix, work left undone, pain management, health education, oral hygiene, documentation, resident rounds, end-of-life support, socialising with the residents to provide stimulation and prevent isolation

1. Tell me about your role in the facility; what tasks do you perform in a workday?

**Probing words:** resident care, resident documentation, resident education, meal assistance, non-nursing duties: housekeeping, cleaning, day shift and night shifts, staff supervision, staff training, scope of practice (within/below/beyond)

Thank you for your participation

**Caregivers**

**Title:** Developing a framework to inform staffing models for long-term care facilities in resource-constrained contexts

**Introduction:** Through the years of coming in close contact with long-term care facilities, I became interested in how the legally prescribed staffing model is implemented in the facilities. Literature showed that staffing shortages in LTCFs occur worldwide and may be influenced by the higher demand for health care due to the growing number of older people. Therefore, the interview guide questions will explore what may help or not help implement the staffing model in the LTCFs. The staffing model refers to the number of nurses and caregivers and how many of each group, as well as how caregivers and nurses are assigned during a typical workday. Your input will be valuable in developing a framework to inform staffing models for long-term care facilities in resource-constrained contexts.

**Questions**

1. Please tell me how you feel about or see the number of caregivers and nurses your LTCF has?

**Probing words**: balance between nurses and caregivers, decision making, resident outcomes

1. In your experience, what helps that you have enough caregivers and nurses in your LTCF?
2. In your experience, what hinders your LTCF from having enough caregivers and nurses?

**Probing words** (questions 2 and 3): recruitment, appointment practices, absenteeism, staff turnover, availability of temporary staff, efforts to adjust staff totals during sick and vacation leave periods

1. Tell me your experience/view on how the nurses and caregivers are allocated to residents or tasks and how the skill mix influences the provision of care?

**Probing words:** who is responsible for the allocation, type of allocation, reasons/bases for distribution, rotating between residents, residents’ acuity, efforts to adjust per residents’ acuity, task allocation, consideration of the scope of practice and skill mix, work left undone, pain management, health education, oral hygiene, documentation, resident rounds, end-of-life support, socialising with the residents to provide stimulation and prevent isolation

1. Tell me about your work in the facility; what jobs do you do in a typical workday?

**Probing words:** resident care, resident documentation, resident education, meal assistance, non-care tasks: housekeeping, cleaning, day shift and night shifts, job description (within / below / beyond), skill mix

**Thank you for your participation**
